# Shorter sleep, higher odds: a nationally representative analysis of the dose-dependent link to depression among 48.8 million U.S. youth

**DOI:** 10.3389/fped.2026.1756808

**Published:** 2026-03-27

**Authors:** Wei Cheng, Shuqi Zhang, Mingjie Shi, Yi Chen, Yi Shi, Junyu Xu

**Affiliations:** 1 Department of Pediatrics, The First Affiliated Hospital of Wannan Medical College, Wuhu City, China; 2 Department of Pediatrics, The First Affiliated Hospital of Huzhou University, Huzhou First People’s Hospital, Huzhou, Zhejiang, China; 3 School of Medicine, Huzhou University, Huzhou, Zhejiang, China

**Keywords:** adolescence, depression, dose-response, epidemiology, inflection point, sleep duration

## Abstract

**Objective:**

While short sleep is linked to depression in youth, the precise dose-response relationship and age-specific risk thresholds remain poorly defined. We aimed to characterize this association and identify optimal sleep durations across development in a nationally representative sample.

**Methods:**

Cross-sectional data from the 2020–2023 National Survey of Children's Health included 126,407 youth aged 6–17 years. Caregiver-reported sleep duration was modeled as a continuous variable using restricted cubic splines and as a categorical variable. Current depression was defined by caregiver-reported clinician diagnosis. Survey-weighted logistic regression adjusted for sociodemographic and health covariates.

**Results:**

A strong, non-linear inverse association was observed between sleep duration and depression risk (*P*-non-linearity <0.001). The dose-response curve plateaued at ∼10 h for ages 6–9, approximately 8.5 h for male aged 10–13, and ∼8.3 h for ages 14–17. Each additional hour of sleep up to these thresholds was associated with significantly reduced odds of depression. The association was strongest in children without ADHD, those in better health, and females (*P*-interaction <0.05). Mediation via the tested pathways (household income, caregiver mental health, child general health, ADHD diagnosis, and caregiver education) accounted for <4% of the total effect.

**Conclusion:**

This study identifies developmentally specific sleep thresholds beyond which the protective effect on depression risk plateaus. These findings provide empirical benchmarks for age-graded sleep recommendations and mental health screening strategies in pediatric populations.

## 1 Introduction

Adequate sleep is fundamental to healthy neurodevelopment and emotional regulation during childhood and adolescence, stages marked by the rapid maturation of prefrontal–limbic circuitry that heightens susceptibility to sleep-related disruptions in mood and cognitive function (1, 2). A substantial body of epidemiological research confirms that insufficient sleep is highly prevalent—affecting nearly two-thirds of U.S. middle and high school students—and consistently associated with a range of adverse mental health outcomes, including depression, anxiety, and behavioral difficulties (3, 4).

Numerous cross-sectional and longitudinal studies report a strong correlation between short sleep duration and elevated depressive symptoms in adolescents (5–7). Meta-analyses further support a directional association in which sleep disturbances often precede the onset of depression, suggesting a potentially causal role (8). In addition, Mendelian randomization and genetic studies provide evidence that genetically proxied short sleep contributes to an increased risk of depression (9). Together, these findings underscore inadequate sleep as a intervention target for mental health disorders across developmental periods.

Despite this evidence, important gaps persist. Many prior studies have treated sleep duration dichotomously (e.g., “short” vs. “adequate”), precluding identification of threshold effects where protective benefits may saturate (10). The functional form of the sleep-depression dose-response remains undefined—whether risk decreases linearly or exhibits nonlinear plateaus—and optimal sleep thresholds across developmental stages are unknown given age-related changes in sleep physiology (11). Application of flexible non-linear modeling (e.g., restricted cubic splines) is therefore required to characterize inflection points and age-specific thresholds.

Moreover, few investigations have utilized large, nationally representative datasets that allow for stratified analyses across diverse demographic and clinical subgroups. The National Survey of Children's Health (NSCH)—an ongoing, cross-sectional survey conducted by the CDC—provides a unique opportunity to address these questions with harmonized measures of sleep, mental health, and sociodemographic factors (12–14).

Accordingly, this study aimed to quantify the dose-dependent relationship between sleep duration and depression in a nationally representative sample of U.S. youth using data from the 2020–2023 NSCH. Applying weighted regression and restricted cubic spline (RCS) models, we sought to: (1) characterize the functional form and inflection points of the sleep–depression association; (2) identify age-specific variations in optimal sleep duration; and (3) evaluate whether key sociodemographic and health-related factors confound, mediate, or moderate the sleep-depression association, informing the generalizability and mechanistic pathways of observed relationships.

## 2 Methods

### 2.1 Data source and study population

Analytical data were extracted from the 2020–2023 cycles of the National Survey of Children's Health (NSCH), a federally mandated, cross-sectional surveillance system that generates annually updated, nationally representative statistics on the physical, emotional, and social well-being of the U.S. pediatric population (0–17 years). Fielded by the Centers for Disease Control and Prevention (CDC), the NSCH employs a complex, address-based sampling frame with stratification by state and oversampling of low-income and racial/ethnic minority children. Information is acquired through standardized caregiver-completed questionnaires that capture granular data on health outcomes (e.g., depression symptomatology), modifiable behaviors (e.g., habitual sleep duration), and a comprehensive array of sociodemographic covariates. All survey procedures are conducted in accordance with the ethical standards of the NSCH Research Ethics Review Board, and written informed consent is secured from each participating caregiver before data collection.

To maximize the developmental validity of the exposure and outcome assessments, the analytic cohort was restricted to children aged 6–17 years. Preschool-aged children (<6 years) were “a priori” excluded because (i) their sleep architecture is characterized by daytime napping and highly variable nocturnal patterns that preclude reliable quantification of “nightly” sleep duration (15), and (ii) caregiver-reported depression screens lack construct validity in this age stratum, introducing non-differential outcome misclassification (16).

**Figure 1:**
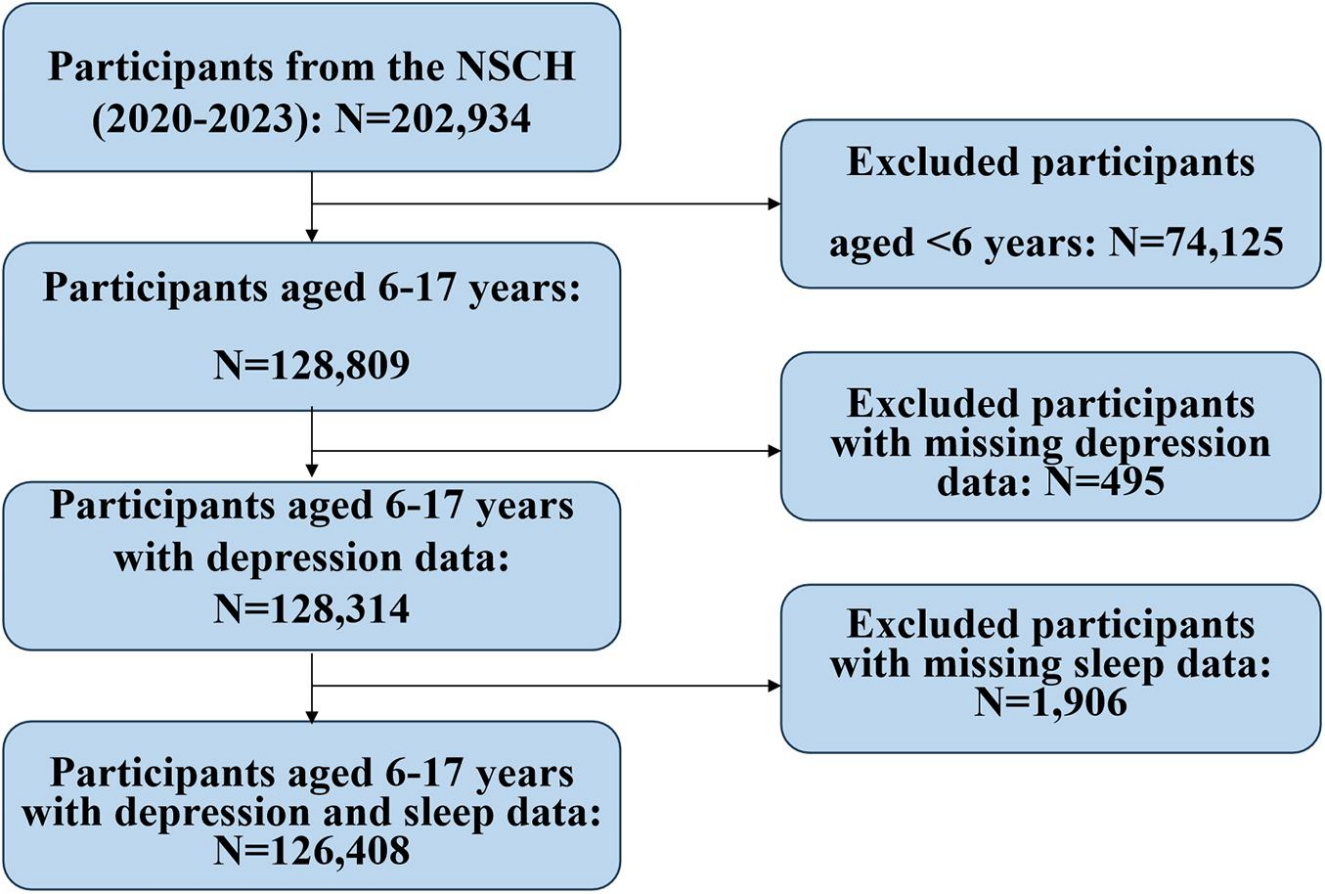
Participant selection flowchart (2020–2023 NSCH). Of 202,934 original participants, 74,125 (age <6 years), 495 (missing depression data), and 1,906 (missing sleep data) were excluded; 126,408 participants aged 6–17 years were finally included.

Sequential application of exclusion criteria yielded the final analytic sample (Figure 1):

1. Enrollment in the 2020–2023 NSCH cycles (*N* = 202,934);
2. Age 6–17 years at interview (excluded *n* = 74,125);
3. Non-missing caregiver-reported depression status (excluded *n* = 495);
4. Non-missing caregiver-reported average nightly sleep hours in the past week (excluded *n* = 1,906).

After exclusions, 126,408 participants remained. Sampling weights that adjust for selection probability, non-response, and post-stratification to the American Community Survey were applied, projecting to a weighted population of 48,766,825 non-institutionalized U.S. children and adolescents aged 6–17 years—thus ensuring that all estimates reflect the contemporary demographic composition of the nation's pediatric population.

### 2.2 Definition of depression and sleep duration

Depression, as operationalized in the present study, refers to caregiver-reported current depressive symptomatology in children and adolescents aged 6–17 years. Consistent with the diagnostic framework of the Diagnostic and Statistical Manual of Mental Disorders, Fifth Edition (DSM-5), depression is characterized by persistent sadness, loss of interest or pleasure, and associated emotional, cognitive, and behavioral impairments that significantly interfere with daily functioning and psychosocial development (17).

The 2020–2023 NSCH captures this construct through two sequential caregiver-reported items: (1) whether the child has ever been diagnosed with depression by a healthcare provider, and (2) whether the child currently has the condition. A child was considered to have current depression if both conditions were affirmed.

Sleep duration was ascertained through a caregiver-reported item that quantified the child's habitual 24-hour sleep time. For children aged 4 months–5 years the question captured total daily sleep (night-time sleep plus daytime naps), whereas for children 6–17 years it referred specifically to sleep obtained on most weeknights. Responses were recorded in integer hours; values <4 h or >16 h were flagged for secondary review against age-specific physiological norms. Values were treated as implausible and set to missing if they fell outside biologically plausible ranges for the specific age group (e.g., <6 h or >12 h for ages 6–9 years, <5 h or >11 h for ages 10–13 years, and <4 h or >10 h for ages 14–17 years), based on established pediatric sleep guidelines and consensus recommendations.

### 2.3 Covariates

Eight covariates, selected *a priori* based on theoretical and empirical relevance to the sleep-depression association, were included. All measures were caregiver-reported. Covariates and their operationalization were as follows: child's gender (female vs. male); age group (6–9, 10–13, or 14–17 years, corresponding to key developmental stages); race/ethnicity (non-Hispanic White, Black, Hispanic, or Other/Multi-racial); household income-to-poverty ratio (FPL) categorized as Poverty (50%–99% FPL), Low-income (100%–199%), Mid-income (200%–299%), or High-income (300%–400%); caregiver-rated mental health (Very poor to Very good); ADHD diagnosis (yes vs. no); caregiver-rated child general health (Poor to Excellent/Very good); and caregiver education (<high school to ≥college degree). All variables had minimal missingness (<0.5%) and were handled with multiple imputations per NSCH guidelines.

### 2.4 Statistical analyses

All analyses accounted for the complex sampling design of the 2020–2023 NSCH using survey weights, strata, and primary sampling units to generate nationally representative estimates. Analyses were performed in R (version 4.5.1) with the survey and srvyr packages. Descriptive statistics are presented as weighted means ± standard error (SE) for continuous variables and weighted proportions (%) with 95% confidence intervals (CI) for categorical variables.

The sleep-depression association was assessed using multivariable logistic regression (svyglm with quasibinomial family). Sleep duration was modeled as a four-level categorical variable and as a continuous variable using RCS with 7 RCS joint point to test for non-linearity. We built three sequential models:

  Model 1: Unadjusted.

  Model 2: Adjusted for age and gender and race/ethnicity.

  Model 3: Additionally adjusted for family income grade, caregiver mental health, ADHD diagnosis, child general health, and caregiver education level.

Results are reported as odds ratios (ORs) with 95% CIs. A trend test was performed by treating the categorical sleep variable as ordinal.

Non-linearity was evaluated by comparing the RCS model to a linear model via a likelihood-ratio test. Stratified analyses were conducted using the fully adjusted Model 3 across pre-specified subgroups. The inflection point—mathematically defined as the sleep duration at which the second derivative is maximized (i.e., where the slope changes most markedly)—was identified to represent the onset of the plateau effect. Effect modification was assessed using Wald tests for interaction terms, with a *P* for interaction <0.05 considered significant.

We conducted a weighted mediation analysis to estimate the proportion of the total sleep-depression effect mediated through three biologically plausible variables: caregiver mental health, ADHD diagnosis, and child general health. Household income and caregiver education were retained in the fully adjusted Model 3 as potential confounders (given their temporal precedence and association with both sleep and depression), but due to the lack of a theoretically coherent pathway by which child sleep duration could influence these socioeconomic factors. The analysis used 1,000 bootstrap replicates to calculate bias-corrected 95% CIs for the percentage mediated.

No a-priori power calculation was performed, two-sided *P*-value <0.05 defined statistical significance.

## 3 Results

### 3.1 Study population and baseline characteristics

Table 1 presents the baseline characteristics of the 126,408 study participants, stratified by sleep duration groups. Sleep duration was categorized into four levels based on caregiver report: Group 1 (≤7 h), Group 2 (8 h), Group 3 (9 h), and Group 4 (≥10 h).

**Table 1:** Baseline characteristics of the study population.

Characteristic	[ALL] *N* = 126,408	Group1 *N* = 21,299	Group2 *N* = 42,917	Group3 *N* = 35,485	Group4 *N* = 26,707	p.overall
Depression						<0.001
Yes	10,659 (8.4%)	3,657 (17.2%)	3,510 (8.2%)	2,097 (5.9%)	1,395 (5.2%)	
No	115,749 (91.6%)	17,642 (82.8%)	39,407 (91.8%)	33,388 (94.1%)	25,312 (94.8%)	
Gender						<0.001
Female	60,817 (48.1%)	10,552 (49.5%)	20,415 (47.6%)	16,771 (47.3%)	13,079 (49.0%)	
Male	65,591 (51.9%)	10,747 (50.5%)	22,502 (52.4%)	18,714 (52.7%)	13,628 (51.0%)	
Age						<0.001
10–13	39,468 (31.2%)	4,551 (21.4%)	13,997 (32.6%)	13,439 (37.9%)	7,481 (28.0%)	
14–17	49,357 (39.0%)	14,936 (70.1%)	20,443 (47.6%)	9,723 (27.4%)	4,255 (15.9%)	
6–9	37,583 (29.7%)	1,812 (8.51%)	8,477 (19.8%)	12,323 (34.7%)	14,971 (56.1%)	
Race						<0.001
Black, non-Hispanic	8,494 (6.72%)	2,467 (11.6%)	3,275 (7.63%)	1,613 (4.55%)	1,139 (4.26%)	
Hispanic	18,322 (14.5%)	3,395 (15.9%)	6,649 (15.5%)	4,960 (14.0%)	3,318 (12.4%)	
Other/Multi-racial, non-Hispanic	17,419 (13.8%)	2,908 (13.7%)	6,016 (14.0%)	5,014 (14.1%)	3,481 (13.0%)	
White, non-Hispanic	82,173 (65.0%)	12,529 (58.8%)	26,977 (62.9%)	23,898 (67.3%)	18,769 (70.3%)	
Income_group						<0.001
High-income (300–400%)	68,975 (54.6%)	9,976 (46.8%)	21,775 (50.7%)	20,807 (58.6%)	16,417 (61.5%)	
Low-income (100–200%)	20,892 (16.5%)	4,122 (19.4%)	7,704 (18.0%)	5,358 (15.1%)	3,708 (13.9%)	
Mid-income (200–300%)	20,410 (16.1%)	3,630 (17.0%)	7,132 (16.6%)	5,633 (15.9%)	4,015 (15.0%)	
Poverty (50–100%)	16,131 (12.8%)	3,571 (16.8%)	6,306 (14.7%)	3,687 (10.4%)	2,567 (9.61%)	
Caregiver_mentalhealth						<0.001
Average	29,763 (23.5%)	6,056 (28.4%)	10,087 (23.5%)	7,907 (22.3%)	5,713 (21.4%)	
Good	51,349 (40.6%)	8,243 (38.7%)	17,385 (40.5%)	14,802 (41.7%)	10,919 (40.9%)	
Poor	7,520 (5.95%)	1,896 (8.90%)	2,469 (5.75%)	1,839 (5.18%)	1,316 (4.93%)	
Very good	36,848 (29.2%)	4,832 (22.7%)	12,664 (29.5%)	10,758 (30.3%)	8,594 (32.2%)	
Very poor	928 (0.73%)	272 (1.28%)	312 (0.73%)	179 (0.50%)	165 (0.62%)	
ADHD_Diagnosis						<0.001
Yes	18,961 (15.0%)	4,325 (20.3%)	6,413 (14.9%)	4,822 (13.6%)	3,401 (12.7%)	
No	107,447 (85.0%)	16,974 (79.9%)	36,504 (85.1%)	30,663 (86.4%)	23,306 (87.3%)	
Health_Status						<0.001
Excellent/Very good	80,013 (63.4%)	11,004 (51.8%)	26,800 (62.5%)	23,573 (66.5%)	18,636 (69.9%)	
Fair	10,646 (8.43%)	2,916 (13.7%)	3,700 (8.63%)	2,436 (6.87%)	1,594 (5.98%)	
Good	33,902 (26.9%)	6,778 (31.9%)	11,868 (27.7%)	9,119 (25.7%)	6,137 (23.0%)	
Poor	1,652 (1.31%)	537 (2.53%)	502 (1.17%)	323 (0.91%)	290 (1.09%)	
Caregiver_highest_education_grade						<0.001
College degree or higher	82,422 (65.2%)	12,118 (56.9%)	26,144 (60.9%)	24,479 (69.0%)	19,681 (73.7%)	
High school or GED	21,095 (16.7%)	4,598 (21.6%)	8,272 (19.3%)	5,079 (14.3%)	3,146 (11.8%)	
Less than high school	4,890 (3.87%)	1,119 (5.25%)	1,928 (4.49%)	1,121 (3.16%)	722 (2.70%)	
Some college or technical school	18,001 (14.2%)	3,464 (16.3%)	6,573 (15.3%)	4,806 (13.5%)	3,158 (11.8%)	

The *p*-value was calculated by the weighted linear regression model. (%) for categorical variables: the *p*-value was calculated by the weighted chi-square test.

The cohort had a mean age of 11.9 years, with a near-equal distribution by gender (51.9% male, 48.1% female). The majority of participants (65.0%) were non-Hispanic White, followed by Hispanic (14.5%), and non-Hispanic Black (6.72%). Socioeconomically, over half of the children (54.6%) lived in high-income households (300%–400% FPL), while 12.8% lived in poverty (50%–99% FPL). Most caregivers held a college degree or higher (65.2%) and reported good or very good mental health (69.8%). Regarding health status, the prevalence of an ADHD diagnosis was 15.0%, and most children were reported to be in excellent, very good, or good health (90.3%).

A clear gradient was observed between these sleep duration groups and the prevalence of current depression, which decreased from 17.2% in Group 1 to 5.2% in Group 4 (*p* < 0.001). Similarly, significant distributions were noted across all examined covariates. Older adolescents (14–17 years) were more prevalent in the shorter sleep groups (Group 1 and 2), whereas younger children (6–9 years) dominated the longer sleep groups (Group 3 and 4). Participants with longer sleep were more likely to be non-Hispanic White, come from higher-income households, and have caregivers with higher educational attainment and better mental health. Furthermore, the prevalence of ADHD diagnosis and poorer child health status was notably higher in the groups with shorter sleep duration.

All differences across sleep duration groups were statistically significant (*p* < 0.001 for all), as determined by weighted chi-square tests.

### 3.2 Sleep duration and depression: overall analyses

#### 3.2.1 Logistic regression analysis

Consistent with the univariate gradient shown in Table 1 (*p* < 0.001), multivariable logistic regression revealed a robust, dose-dependent reduction in depression risk across sleep-duration groups (Table 2). Relative to the shortest-sleep group (Group 1), fully adjusted odds ratios (Model 3) were: Group 2: OR = 0.65 (95% CI 0.57–0.73); Group 3: OR = 0.67 (95% CI 0.58–0.77); Group 4: OR = 0.76 (95% CI 0.65–0.89). The slightly attenuated OR in Group 4 (vs. Group 3) was not statistically significant (Wald test *P* = 0.12), consistent with the plateau effect observed in RCS analysis. A significant inverse trend was observed across all models (*p*-trend <0.001).

**Table 2:** Weighted logistic regression analysis table.

GROUP	Model 1 OR(95%CI)	Model 1 *p*-value	Model 2 OR(95%CI)	Model 2 *p*-value	Model 3 OR(95%CI)	Model 3 *p*-value
Group2	0.38 (0.34–0.43)	<0.001	0.51 (0.46–0.57)	<0.001	0.65 (0.57–0.73)	<0.001
Group3	0.28 (0.25–0.32)	<0.001	0.51 (0.45–0.58)	<0.001	0.67 (0.58–0.77)	<0.001
Group4	0.25 (0.22–0.28)	<0.001	0.60 (0.52–0.69)	<0.001	0.76 (0.65–0.89)	0.001
*P* for trend		<0.001		<0.001		<0.001

The weighted logistic regression models were adjusted for all covariates. Odds Ratios (OR) and 95% Confidence Intervals (CI) are reported. *p*-values were derived from the weighted regression models. Model 1: Unadjusted; Model 2: Adjusted for age, gender, and race/ethnicity; Model 3: Adjusted for age, gender, race/ethnicity, household income-to-poverty ratio, caregiver education, caregiver mental health, ADHD diagnosis, and child general health.

#### 3.2.2 Nonlinear dose-response relationship and inflection point

**Figure 2:**
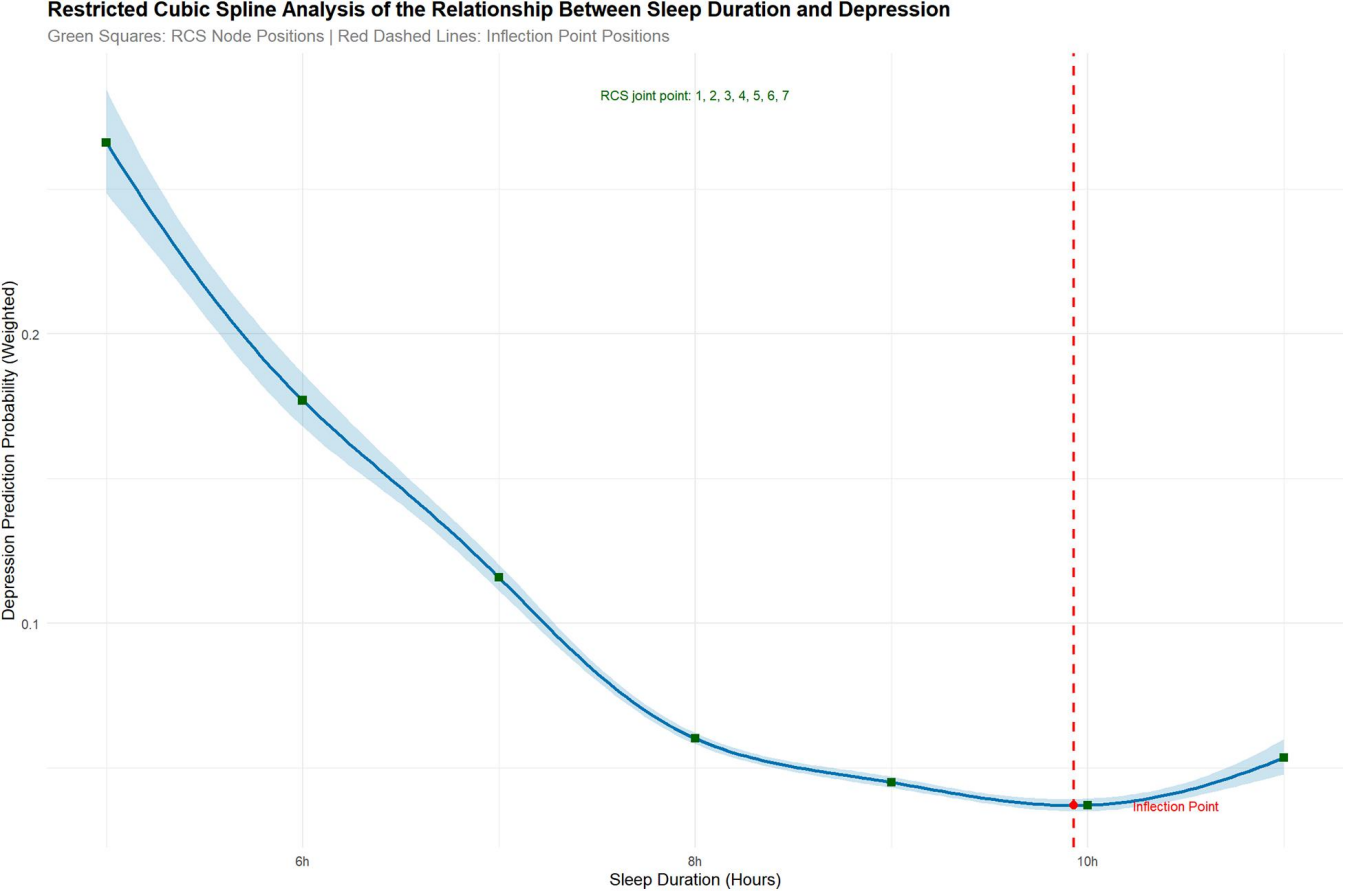
Restricted cubic spline (RCS) of sleep duration vs. depression risk. Green squares = RCS nodes; red dashed line = inflection point (∼10 h). Adjusted for covariates; non-linearity *P* < 0.001. Depression risk decreased with sleep duration until 10 h, then plateaued.

**Figure 3:**
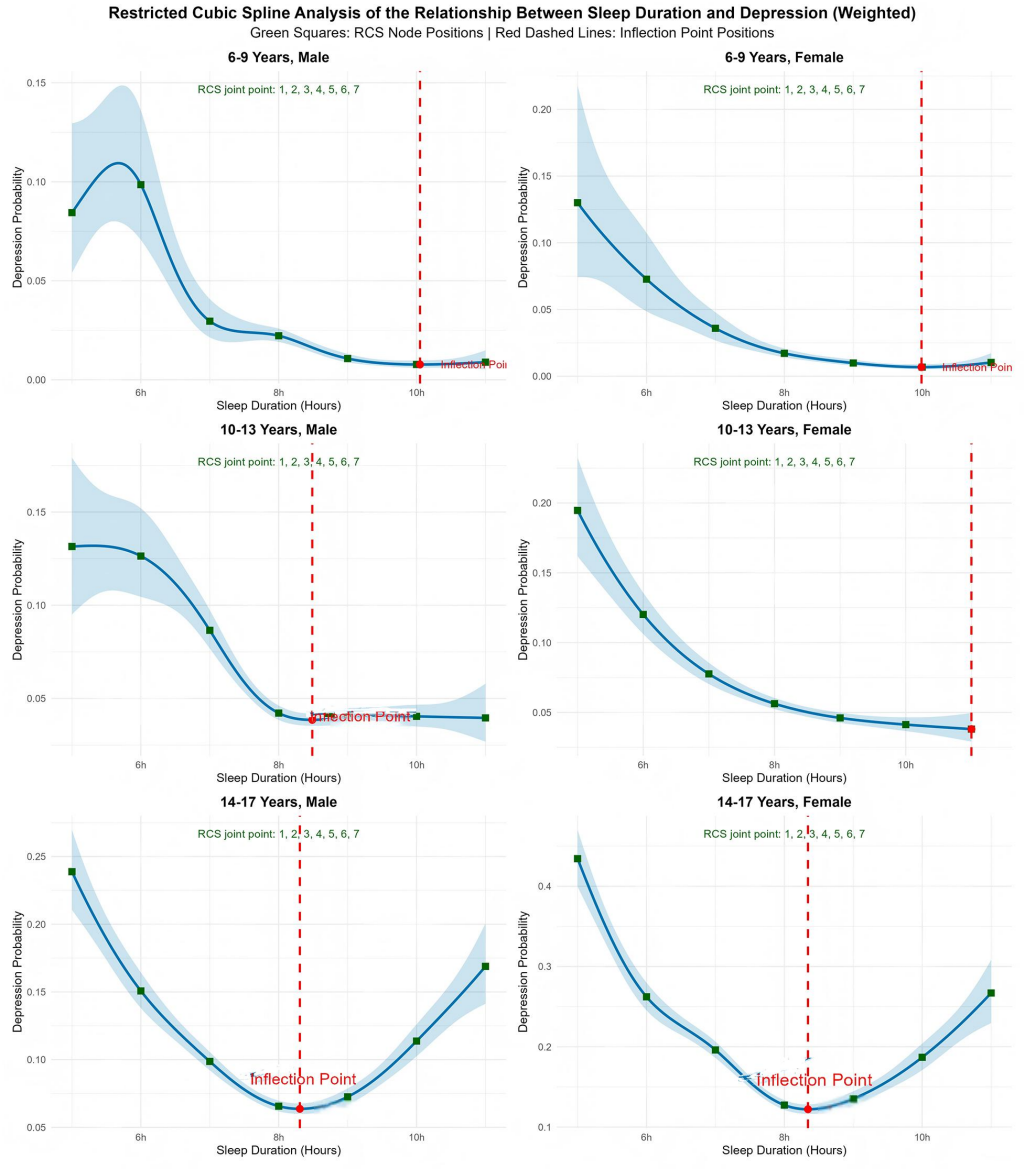
Stratified RCS of sleep duration vs. depression risk (by age/gender). Green squares = RCS nodes; red dashed lines = age-specific inflection points (∼10 h for 6–9 years, ∼8.5 h for 10–13 years, ∼8.3 h for 14–17 years). Non-linearity *P* < 0.001 for all subgroups.

A restricted cubic spline (RCS) analysis was conducted to examine the potential nonlinear dose-response relationship between sleep duration and depression, using the rms package in R. Knots were placed at the 1st, 10th, 25th, 50th, 75th, 90th, and 99th percentiles of sleep duration, this percentile-based placement strategy ensures robust coverage across the full exposure range while balancing model flexibility and stability (indicated by green squares in Figure 2, 3). All models were adjusted for age, gender, race/ethnicity, household income, caregiver mental health, caregiver education, ADHD diagnosis, and general health status. The inflection point—defined as the sleep duration at which the slope of the curve changed most markedly—was identified, along with its 95% confidence interval, using bootstrapping with 1,000 iterations.

In the total population, the RCS model (Figure 2 reveals a distinct nonlinear dose-response curve with a plateau near 10 h, indicating that protective benefits saturate beyond this threshold.) revealed a significant nonlinear association between sleep duration and depression (*P* for nonlinearity <0.001). The curve exhibited a clear inverse relationship, with the predicted probability of depression declining as sleep duration increased. The dose-response curve exhibited a plateau point at approximately 10 h of sleep, beyond which the predicted probability of depression stabilized, indicating a potential saturation of the protective effect. Below this threshold, depression probability decreased steadily; beyond it, the curve plateaued, suggesting no further reduction in depression risk with additional sleep.

Stratified RCS analyses across age and gender subgroups [Figure 3 demonstrates age-specific inflection points (10 h →  8.5 h → 8.3 h) that shift to lower durations with increasing age, mirroring developmental changes in sleep physiology.] consistently showed significant nonlinear associations (*P* for nonlinearity <0.001 for all). However, the inflection points varied by developmental stage:

Ages 6–9: The inflection point occurred at approximately 10 h for both males and females.

Ages 10–13: The inflection point shifted earlier to approximately 8.5 h in males, while no distinct inflection point was observed in females.

Ages 14–17: The inflection point was observed at approximately 8.3 h for both sexes. While the absolute depression risk was highest in this group—particularly among females—the shape of the dose-response curve and inflection point remained consistent.

### 3.3 Sleep duration and depression: subgroup analyses and *P* for interaction

**Figure 4:**
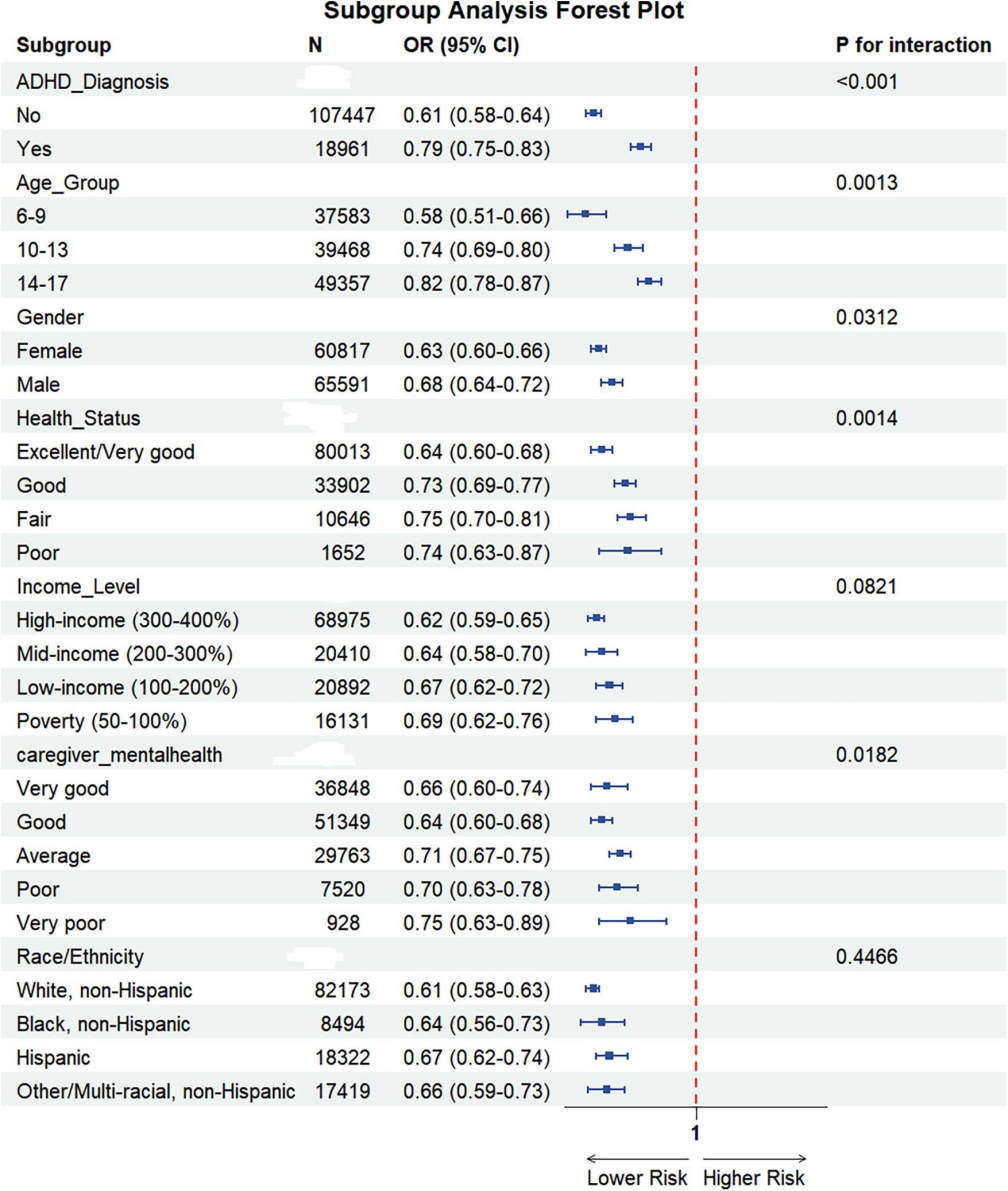
Forest plot of subgroup analyses. Shows OR (95%CI) for sleep duration–depression association across subgroups (ADHD, age, gender, etc.) and *P*-values for interaction, illustrating differential association strengths.

Stratified analyses were performed to evaluate potential effect modification of the sleep-depression association by key demographic and clinical characteristics. A statistically significant interaction was observed for ADHD diagnosis (*P* for interaction <0.001), with a stronger inverse association among children without an ADHD diagnosis (OR = 0.61, 95% CI: 0.58–0.64) compared to those with a diagnosis (OR = 0.79, 95% CI: 0.75–0.83). (Table 3; Figure 4 illustrates the attenuated association in adolescents and those with ADHD, highlighting differential vulnerability across subgroups.)

**Table 3:** Subgroup analysis table.

Subgroup	OR(95%CI)	P_value	N	*P* for interaction
ADHD_Diagnosis				<0.001
No	0.61 (0.58–0.64)	<0.001	107,447	
Yes	0.79 (0.75–0.83)	<0.001	18,961	
Age_Group				0.001
6–9	0.58 (0.51–0.66)	<0.001	37,583	
10–13	0.74 (0.69–0.80)	<0.001	39,468	
14–17	0.82 (0.78–0.87)	<0.001	49,357	
Gender				0.031
Female	0.63 (0.60–0.66)	<0.001	60,817	
Male	0.68 (0.64–0.72)	<0.001	65,591	
Health_Status				0.001
Excellent/Very good	0.64 (0.60–0.68)	<0.001	80,013	
Good	0.73 (0.69–0.77)	<0.001	33,902	
Fair	0.75 (0.70–0.81)	<0.001	10,646	
Poor	0.74 (0.63–0.87)	<0.001	1,652	
Income_Level				0.082
High-income (300–400%)	0.62 (0.59–0.65)	<0.001	68,975	
Mid-income (200–300%)	0.64 (0.58–0.70)	<0.001	20,410	
Low-income (100–200%)	0.67 (0.62–0.72)	<0.001	20,892	
Poverty (50–100%)	0.69 (0.62–0.76)	<0.001	16,131	
Caregiver_mentalhealth				0.018
Very good	0.66 (0.60–0.74)	<0.001	36,848	
Good	0.64 (0.60–0.68)	<0.001	51,349	
Average	0.71 (0.67–0.75)	<0.001	29,763	
Poor	0.70 (0.63–0.78)	<0.001	7,520	
Very poor	0.75 (0.63–0.89)	0.001	928	
Race/Ethnicity				0.447
White, non-Hispanic	0.61 (0.58–0.63)	<0.001	82,173	
Black, non-Hispanic	0.64 (0.56–0.73)	<0.001	8,494	
Hispanic	0.67 (0.62–0.74)	<0.001	18,322	
Other/Multi-racial, non-Hispanic	0.66 (0.59–0.73)	<0.001	17,419	

In the subgroup analysis, the model is not adjusted for the stratification variable itself.

A significant effect modification was also found by age group (*P* for interaction = 0.0013), with the strength of the protective association exhibiting graded attenuation across age strata (OR 0.58 → 0.74 → 0.82) despite inflection points shifting to lower sleep durations. The strength of the inverse association exhibited a graded attenuation with increasing age, being strongest in children aged 6–9 years (OR = 0.58, 95% CI: 0.51–0.66), moderate in those aged 10–13 years (OR = 0.74, 95% CI: 0.69–0.80), and weakest, though still significant, in adolescents aged 14–17 years (OR = 0.82, 95% CI: 0.78–0.87).

Furthermore, significant interactions were identified for child's health status (*P* for interaction = 0.0014) and caregiver mental health (*P* for interaction = 0.0182). The protective association was most pronounced in children with excellent/very good health (OR = 0.64, 95% CI: 0.60–0.68) compared to those with poorer health statuses, and among children of caregivers reporting good mental health (OR = 0.64, 95% CI: 0.60–0.68). A modest but significant interaction was also detected for gender (*P* for interaction = 0.0312), with a slightly stronger association in females (OR = 0.63, 95% CI: 0.60–0.66) than in males (OR = 0.68, 95% CI: 0.64–0.72).

In contrast, no significant effect modification was observed for household income level (*P* for interaction = 0.0821) or race/ethnicity (*P* for interaction = 0.4466), although a trend towards a weaker association was noted with decreasing income levels. A significant inverse association between sleep duration and depression was consistently present across all strata of these subgroups (all *P* < 0.001).

### 3.4 Mediation analysis

We conducted a formal mediation analysis to quantify the extent to which the total effect of sleep duration on depression was explained by three biologically plausible mediators: caregiver mental health, ADHD diagnosis, and child general health status. Household income and caregiver education were incorporated as confounding variables in the fully adjusted model (given their temporal precedence and associations with both sleep duration and depression) rather than as mediators, as there is no theoretically coherent pathway by which child sleep duration could causally influence these socioeconomic factors.

As detailed in Table 4, a significant total effect of sleep duration on depression was consistently observed (*β* = −0.204, *p* < 0.001 for all pathways). The direct effect of sleep on depression remained statistically significant after accounting for all mediators (*p* < 0.001), indicating that the association was not fully explained by the variables tested.

**Table 4:** Weighted mediation analysis.

Mediator/Confounding variables	Total_Effect	*p*-value_Total	Direct_Effect	*p*-value_Direct	Indirect_Effect	*p*-value_Indirect
Income_Level	−0.2037	<0.001	−0.1844	<0.001	−0.0017	<0.001
Caregiver_mentalhealth	−0.2037	<0.001	−0.1348	<0.001	−0.0043	<0.001
ADHD_Diagnosis	−0.2037	<0.001	−0.1745	<0.001	−0.0019	<0.001
Health_Status	−0.2037	<0.001	−0.1353	<0.001	−0.0045	<0.001
Caregiver_highest_grade	−0.2037	<0.001	−0.1956	<0.001	−0.0007	<0.001

Weighted mediation analysis of the effect of sleep duration on depression through five selected variables. Total, direct, and indirect effects are expressed as *β* coefficients; all *p*-values are derived from weighted bootstrap models.

**Figure 5:**
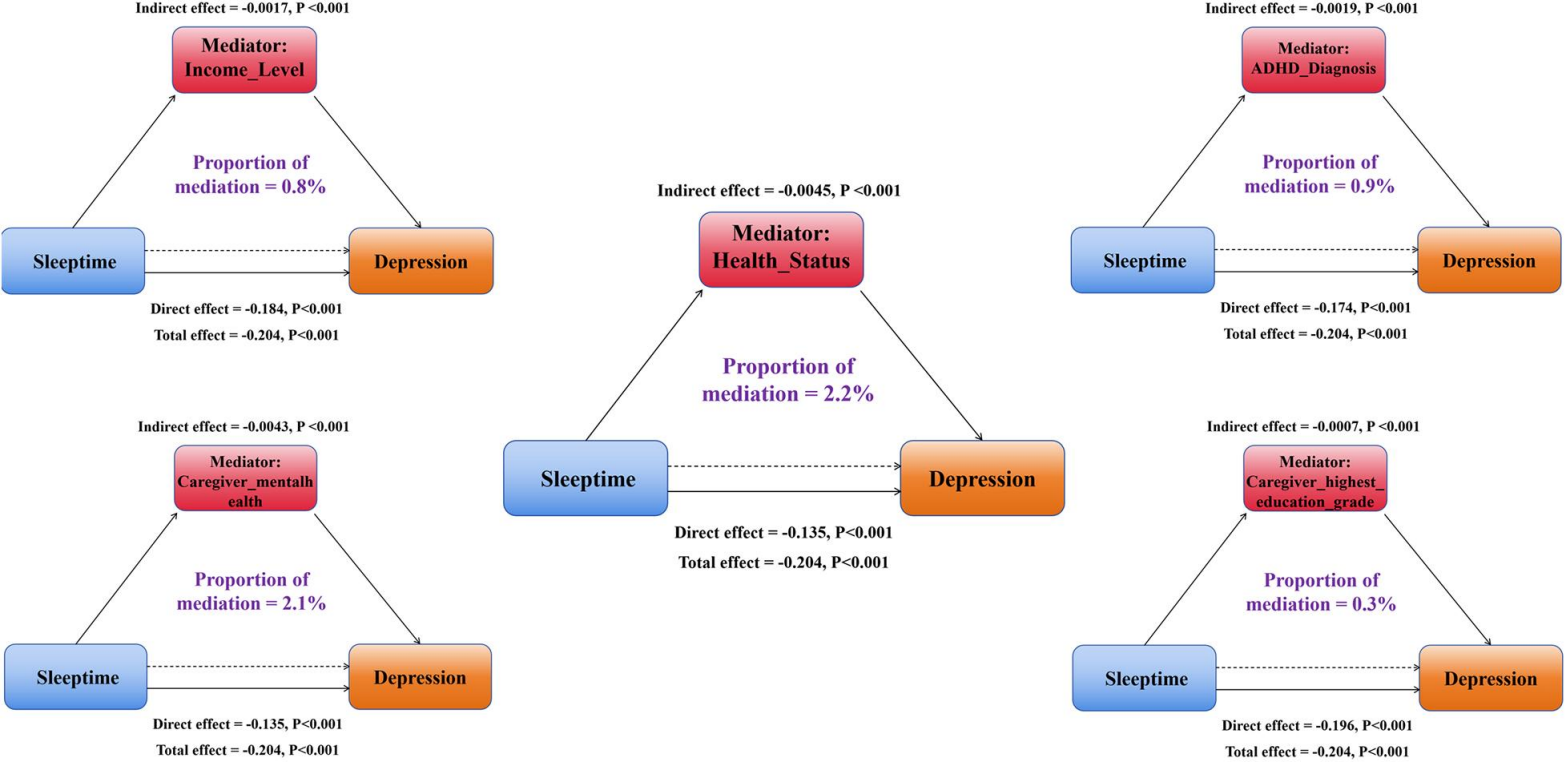
Mediation analysis of sleep duration–depression association. Presents total/direct/indirect effects and mediation proportions for 5 mediators and confounding variables (income, ADHD, health status, etc.). All effects *P* < 0.001; child health and caregiver mental health had relatively larger mediation roles.

However, all indirect effects were also statistically significant (*p* < 0.001), though the proportion of the total effect mediated was modest for all variables [Figure 5 confirms that socioeconomic and health factors explain minimal variance (<4%), supporting a direct biological pathway]. Child general health status (Proportion Mediated = 2.2%) and caregiver mental health (Proportion Mediated = 2.1%) emerged as the most substantial mediators among those examined. In contrast, household income (0.8%), ADHD diagnosis (0.9%), and caregiver education (0.3%) accounted for only a small fraction of the total association.

## 4 Discussion

Using a weighted, nationally representative sample of 48.8 million U.S. children and adolescents from the 2020–2023 National Survey of Children's Health, the present study provides robust population-level evidence of a dose-dependent, inverse association between sleep duration and depression risk. Across fully adjusted models, each incremental hour of sleep was consistently associated with a lower likelihood of current depression, exhibiting a clear gradient from ≤7 h to ≥10 h. The RCS analyses further revealed a significant nonlinear dose–response pattern, characterized by a pronounced decline in depression probability with increasing sleep up to approximately 10 h, beyond which the risk plateaued. This inflection point varied developmentally, shifting from ∼10 h in younger children (6–9 years) to ∼8.3 h in older adolescents (14–17 years), suggesting age-specific sleep requirements for optimal mental health. Subgroup analyses demonstrated that this inverse association persisted across demographic and clinical strata but was strongest among children with better general health and without ADHD, and slightly more pronounced in females than males. Mediation modeling indicated that only a small fraction of the total effect—approximately 2%–4%—was indirectly explained by caregiver mental health and child general health status, reinforcing that the sleep–depression association is predominantly direct rather than those confounding. Collectively, these findings extend existing epidemiologic evidence by delineating a quantitative, nonlinear, and developmentally dynamic pattern linking habitual sleep duration to depression risk in youth, providing empirical thresholds for potential preventive interventions and policy guidance at the population level (18, 19).

Consistent with prior evidence linking short sleep to elevated depression risk (6, 20–22), our study extends this literature by quantifying nonlinear thresholds (10h → 8.3 h) across development, demonstrating graded risk accumulation that saturates rather than extending linearly (23). Notably, whereas prior studies predominantly dichotomized sleep (e.g., <8 h vs. ≥8 h)—thereby obscuring threshold effects and assuming linear risk below arbitrary cutoffs (24, 25) —our continuous RCS approach uniquely identified nonlinear inflection points and saturation thresholds. This methodological advance enables precise, age-specific sleep recommendations (e.g., 10 h for ages 6–9 vs. 8.3 h for ages 14–17) that dichotomous classifications cannot provide (26). This plateau pattern has not been demonstrated at a nationally representative, pediatric population scale. Furthermore, our analysis indicates that the inflection point shifts developmentally, decreasing from approximately 10 h in early childhood to ∼8.3 h in mid-to-late adolescence. These results support the hypothesis that adolescent maturation is accompanied by reduced optimal sleep thresholds for emotional well-being, which may reflect biological (pubertal neuroendocrine changes), behavioral (academic load, technology use), and psychosocial transitions (27, 28). In addition, the present study fills a key evidence gap by rigorously characterizing dose-dependent sleep–depression relationships among school-aged children (6–9 years), a group typically excluded from prior models due to measurement limitations. Our findings demonstrate that younger children experience the largest relative benefit per unit of additional sleep, emphasizing the developmental salience of sufficient sleep in early life (29).

The minimal mediation observed (<4% via tested health and socioeconomic factors) indicates that the sleep-depression association operates primarily through direct neurobiological mechanisms rather than indirect behavioral pathways. Specifically, short sleep hyperactivates the HPA axis and elevates cortisol (30, 31), disrupts prefrontal cortex-mediated emotion regulation (32), and reduces serotonergic/dopaminergic signaling (33, 34). —processes directly implicated in depression pathophysiology. The negligible contribution of child general health (∼2.2%) and caregiver mental health (∼2.1%) supports the interpretation that sleep loss affects mood regulation through immediate biological perturbations rather than through deterioration of general health status or social functioning, positioning sleep duration as a direct upstream determinant of neurobiological vulnerability (35, 36).

The magnitude of the sleep–depression association varied meaningfully across developmental and clinical subgroups. The strongest inverse association was observed among children aged 6–9 years (OR = 0.58), suggesting heightened vulnerability during a critical window of neurodevelopment. In contrast, the relationship was attenuated among adolescents aged 14–17 years (OR = 0.82). This pattern may reflect the increasing influence of distal psychosocial and hormonal factors—such as peer pressure and academic demands—that dilute the relative contribution of sleep duration to depression risk. A modest sex difference was also detected, with females exhibiting a slightly stronger protective association (OR = 0.63 vs. 0.68 in males), consistent with prior evidence suggesting that girls are more susceptible to sleep-related mood disturbance (37). This may relate to hormonal modulation of circadian processes, including estrogenic effects on sleep timing and homeostatic drive, which could amplify emotional consequences of sleep loss (38). The association was weaker among youth with ADHD (OR = 0.79), a pattern that may arise from co-occurring sleep disturbances—such as prolonged sleep latency or irregular sleep–wake patterns—that limit the incremental benefit of longer sleep duration. This attenuation highlights the need for individualized sleep interventions in neurodevelopmentally affected populations, potentially combining behavioral strategies with pharmacologic therapy. Furthermore, stronger associations in children with better general health suggest that diminished physiological reserve may blunt sleep's protective effects, underscoring the importance of early, holistic approaches to mental health promotion (39).

These findings support the development of age-specific sleep recommendations, suggesting ≥10 h for children aged 6–9 years, ≥8.5 h for those aged 10–13 years, and ≥8.3 h for adolescents aged 14–17 years, consistent with the inflection points identified herein. At the population level, delaying school start times—particularly in middle and high schools—may represent an effective structural strategy, aligning with American Academy of Pediatrics guidance advocating for start times of 8:30 AM or later (40, 41). Integration of sleep duration into routine pediatric and mental health screening may facilitate early identification of vulnerable individuals, especially females, youth with ADHD, and those from structurally disadvantaged households (42). Community-based and family-centered interventions that promote sleep hygiene—such as limiting pre-bedtime screen exposure, which can delay melatonin onset—may further mitigate risk (43). However, several limitations warrant consideration. First, the cross-sectional design precludes causal inference. Second, both sleep duration and depression status relied on caregiver reports, introducing potential shared method variance and recall bias; objective measures (e.g., actigraphy) are needed to validate sleep estimates, and caregiver-reported depression may under- or over-estimate clinical diagnoses. Third, residual confounding is possible given the absence of key behavioral exposures such as social media use and caffeine intake.

These age-specific thresholds carry substantial public health implications. Current guidelines often apply uniform sleep recommendations across broad pediatric age ranges, potentially misaligning with developmental shifts in sleep physiology identified here. By establishing empirically-derived benchmarks (10h → 8.3 h), our findings enable precision prevention strategies—such as age-graded school start times and targeted screening protocols—that account for differential vulnerability across childhood and adolescence, optimizing resource allocation for mental health promotion at the population level.

## 5 Conclusion

In a nationally representative pediatric population, dose–response analyses establish age-specific sleep thresholds (≈10 h at 6–9 y, ≈8.3 h at 14–17 y) above which depression risk plateaus, underscoring sleep as a quantifiable, developmentally modifiable prevention target. These data call for integrating precise, age-graded sleep guidelines into youth mental-health policy and clinical screening.

## Data Availability

The study data are available through the National Survey of Children's Health (NSCH) study webpage (https://mchb.hrsa.gov/data-research/national-survey-childrens-health).
